# An XMRV Derived Retroviral Vector as a Tool for Gene Transfer

**DOI:** 10.1186/1743-422X-8-284

**Published:** 2011-06-08

**Authors:** Daniel Cervantes-Garcia, Augusto Rojas-Martinez, David Camerini

**Affiliations:** 1 Division of Infectious Diseases, Center for Virus Research and Institute for Immunology, University of California, Irvine, California, 92697-4068, USA; 2 Department of Biochemistry and Molecular Medicine in the School of Medicine, Centro de Investigacion y Desarrollo en Ciencias de la Salud, Unidad de Terapia Genica y Celular, Universidad Autonoma de Nuevo Leon, Monterrey, Nuevo Leon, Mexico

**Keywords:** XMRV, retroviral vector, transduction

## Abstract

**Background:**

Retroviral vectors are widely used tools for gene delivery and gene therapy. They are useful for gene expression studies and genetic manipulation *in vitro *and *in vivo*. Many retroviral vectors are derived from the mouse gammaretrovirus, murine leukemia virus (MLV). These vectors have been widely used in gene therapy clinical trials. XMRV, initially found in prostate cancer tissue, was the first human gammaretrovirus described.

**Findings:**

We developed a new retroviral vector based on XMRV called pXC. It was developed for gene transfer to human cells and is produced by transient cotransfection of LNCaP cells with pXC and XMRV-packaging plasmids.

**Conclusions:**

We demonstrated that pXC mediates expression of inserted transgenes in cell lines. This new vector will be a useful tool for gene transfer in human and non-human cell lines, including gene therapy studies.

## Findings

Retroviral vectors offer a highly efficient method of stable gene transfer in mammalian cells due to their ability to integrate into the host genome [1,2]. Moreover, the common genetic architecture of most retroviruses allows the development of similar retroviral vectors with different potentials for cell entry via virus-specific receptors and different capabilities for gene expression mediated by diverse retroviral promoters [3].

Current retroviral vectors used for gene transfer are replication defective. Trans-expression of retroviral structural proteins from non vector-homologous plasmids avoids the production of replication competent retrovirus (RCR) [4,5]. Many retroviral vectors are derived from murine leukemia virus (MLV) in both, ecotropic and amphotropic versions [6,7]. Lentiviral vectors based on HIV may offer advantages because of their lower insertion frequency in crucial *loci *involved in cell growth regulation and their ability to transduce non-dividing cells [8,9]. Nevertheless, MLV-derived retroviral vectors have been used extensively, including in more than 300 gene therapy clinical trials [10]. In addition, retroviral vectors derived from avian sarcoma leukosis virus (ASLV; [11]), spleen focus-forming virus (SFFV; [12]), and Mason-Pfizer monkey virus (MPMV; [13]), have been developed among others.

In 2006, the xenotropic murine-leukemia-virus related gammaretrovirus (XMRV) was discovered in a subset of human prostate cancer (PCa) tissue samples [14]. Subsequently, additional studies demonstrated that XMRV uses the XPR1 receptor to initiate infection, and that the virus is sensitive to IFN-β and RNase-L, a final effector of the IFN-β mediated antiviral response [15]. Since XMRV shows the basic structure of gammaretroviruses, we developed a panel of packaging plasmids and retroviral vectors derived from XMRV. Here we demonstrate their potential use as gene transfer vectors for *in vitro *assays.

**Figure 1:**
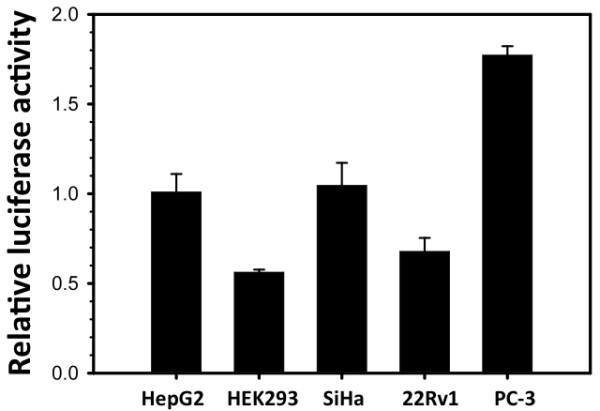
**Promoter activity of XMRV U3 region**. The pGL-XMRV-U3-Luc plasmid was used to transfect five cell lines and luciferase activity was compared to the activity of cells transfected with pGL3 SV40-Luc. Data are represented as the mean of triplicates ± standard error (s.e.).

Initially, we constructed a plasmid to evaluate the promoter activity of the U3 region of the XMRV LTR derived from 22Rv1 cells. A fragment of 554 base pairs was isolated by PCR with oligonucleotide primers XMRV-U3-f and XMRV-U3-r (Table 1), and inserted into the plasmid vector, pCR2.1-Topo (Invitrogen). Digestion with the enzymes *Sac-*I and *Xho-*I released a fragment of 632 base pairs, which was inserted into the plasmid vector, pGL3-Basic (Promega). The pGL3-XMRV-U3-luc expression plasmid was used to transfect Hep G2, HEK-293, SiHa, 22Rv1, and PC-3 cell lines. Luciferase expression was assayed 72 hours later. Similar levels of transcription were observed in all five cell lines tested and little or no cell type specificity of the XMRV promoter was found. Moreover, the level of luciferase expression mediated by the XMRV promoter was similar to that mediated by the SV40 virus early region promoter in each cell line (Figure 1).

**Table 1:** Oligonucleotide primers used in this study.

Oligonucleotide	5' modification	Sequence
**Luciferase expression plasmid**		
XMRV-U3-f	-	GCCCTGGTTCTGACCCAACAGTAT
XMRV-U3-r	-	AAAGGCTTTATTGGGAACACGGGT

**Vector plasmid**		
CMV-IEE-f	*Sac*I	GAGCTCCGCGTTACATAACTTACGG
CMV-IEE-r	*Mfe*I	CAATTGCAAAACAAACTCCCATTGACG
XMRV-LTR5-f	*Mfe*I	CAATTGTGAAAGACCCCACCATAAGG
XMRV-Δgag-r	*Avr*II	CCTAGGACGATCCCGAGAACCGTAAC
CMV-IEP-f	*Avr*II	CCTAGGGTTGACATTGATTATTGAC
CMV-IEP-r	*Xho*I	CTCGAGGTCTGCTTATATAGACC
XMRV-PPT-LTR3-f	*Xba*I	TCTAGAATTTCGGTAGTGCAGGCCCTGG
XMRV-PPT-LTR3-r	*Spe*I	ACTAGTAATGAAAGACCCCCGAGCTGGG

**Packaging plasmid gag-pol**		
XMRV-gagpol-f	*Nhe*I	GCTAGCATCATGGGACAGACCGTAACTAC
XMRV-gagpol-r	*Not*I	GCGGCCGCTTAGGGAAAGTGTCTGTCATCGT

**Packaging plasmid env**		
XMRV-env-f	*Kpn*I	GGTACCCATGGAAATGCCAGCGTTCTCAA
XMRV-env-r	*Not*I	GCGGCCGCGCTAGCGTGCTAAGCCTTAT

**Figure 2:**
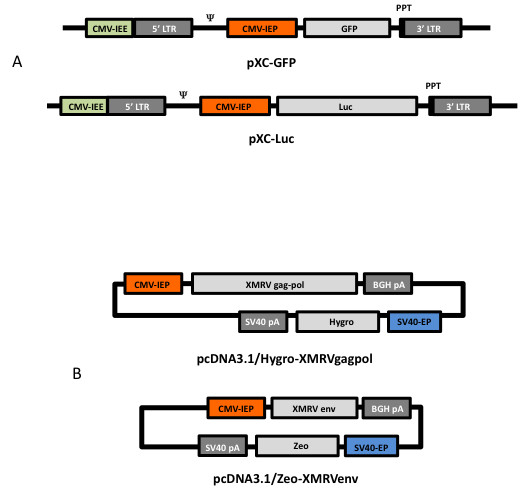
**Vectors and packaging plasmids**. Schematic maps of (*A*) XMRV-derived vectors, and (*B*) XMRV vector packaging plasmids.

An XMRV-derived retroviral gene transfer vector, pXC, was constructed in the pBluescript II KS plasmid. Genomic DNA from 22Rv1 cells was used as template for XMRV sequence isolation by PCR amplification. The CMV immediate early enhancer (CMV-IEE) was positioned 5' to the XMRV LTR and XMRV RNA-packaging signal, followed by the CMV immediate early promoter (CMV-IEP) driving a reporter gene (GFP or luciferase), the XMRV polypurine tract, and a 3' XMRV LTR (Figure 2). GFP or luciferase genes were inserted in the *Xho-*I/*Sal-*I and *Xba-*I sites. XMRV vector packaging plasmids pcDNA3.1/Hygro-XMRV*gagpol *and pcDNA3.1/Zeo-XMRV*env *were also created by amplification of the *gag-pol *and *env *genes, respectively. The templates for isolation of XMRV structural genes were the VP62 clone [14] for the *gag-pol *gene, and genomic DNA from the 22Rv1 line for the *env *gene. Primers used for these steps are described in Table 1.

**Figure 3:**
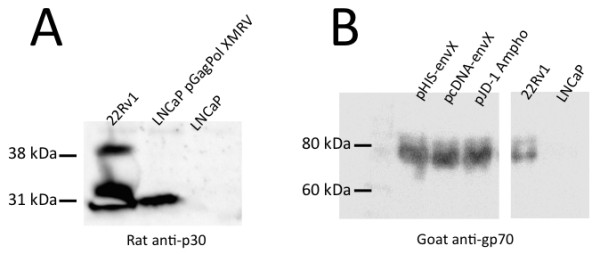
**XMRV structural proteins**. Western blots were performed for detection of the p30 capsid subunit of Gag and the gp70 surface glycoprotein subunit of Env in LNCaP cells.

XMRV structural proteins were expressed in LNCaP cells, since these cells satisfactorily support XMRV protein expression and replication [16,17]. LNCaP cells were transfected with the packaging plasmids (50 μg each) using Lipofectamine and XMRV Gag and Env protein expression was detected by SDS-PAGE followed by western blotting (Figure 3). Ninety-six hours post-transfection, cells were harvested and proteins were extracted with lysis buffer (1% Triton X-100, 10 mM Tris-HCl pH 7.5, 50 mM KCl, 2 mM MgCl_2_, 0.5 mM PMSF, 0.02 mM β-mercaptoethanol, and protease inhibitor cocktail). The XMRV capsid protein (p30) and surface glycoprotein (gp70) were detected using rat monoclonal anti-p30 antibody (kindly provided by Drs. Sandra and Francis Ruscetti), goat anti-gp70 serum (kindly provided by Dr. John Elder), goat anti-rat IgG conjugated to horseradish peroxidase (HRP; Invitrogen), and chicken anti-goat IgG conjugated to HRP (Santa Cruz Biotech) respectively. HRP activity was detected by chemiluminescence using a commercial kit (Pierce).

**Figure 4:**
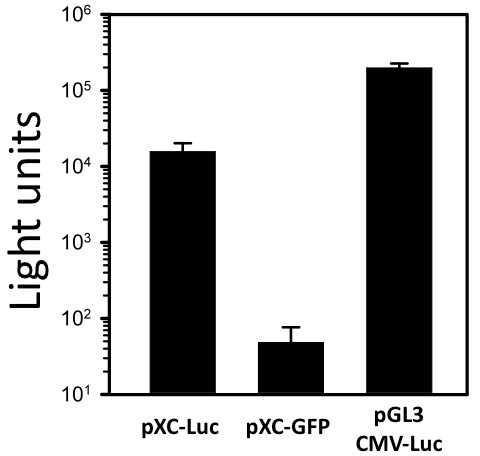
**Gene expression mediated by XMRV-derived vector in LNCaP cells**. Gene transfer vector XC-Luc or XC-GFP were used to transduce LNCaP cells. Luciferase activity was measured 72 hours after transduction. As a positive control, LNCaP cells were transfected with the plasmid pGL3 CMV-Luc using Lipofectamine. Data are represented as the mean of triplicates ± standard error (s.e.).

Lipofectamine mediated triple transfection of LNCaP cells with the XMRV-derived vector pXC-Luc or pXC-GFP and two XMRV vector packaging plasmids, pcDNA3.1/Hygro-XMRV*gagpol *and pcDNA3.1/Zeo-XMRV*env*, were used to produce XMRV-derived retroviral vectors in the culture media. Cell free supernatants were filtered 72 hours after transfection to produce XMRV vector stocks. Vector stocks were centrifuged onto LNCaP cells at 2500 RPM for 90 minutes at room temperature (spinfected) in the presence of 8 μg/mL polybrene. Seventy-two hours later, luciferase expression in the spinfected LNCaP cells was assayed with a commercial assay kit (Dual-Glo Luciferase Assay System, Promega). The level of luciferase expression achieved in LNCaP cells transduced with pXC-Luc was more than 320-fold greater than the background expression following transduction with pXC-GFP (Figure 4). Luciferase expression mediated by pXC-Luc, however, was lower than expression in LNCaP cells transfected with pGL3 CMV-Luc.

**Figure 5:**
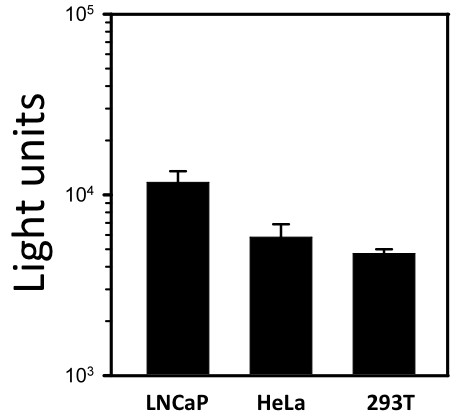
**Transduction of different cell lines with the XMRV-derived vector**. Luciferase activity in LNCaP, HeLa, and 293T cell lines 72 hours after transduction with the XMRV-derived vector, pXC-luc. Data are represented as mean of triplicates ± standard error (s.e.).

293T and HeLa cells were also transduced with pXC-Luc and these cells were analyzed for luciferase expression 72 hours later along with LNCaP cells (Figure 5). Consistent with previous reports, HeLa and 293T cells were transduced with pXC, but luciferase expression was lower than in LNCaP cells.

## Conclusions

Many retroviral vectors for gene transfer are derived from murine gamma-retroviruses. Nevertheless, to our knowledge, the pXC vector and its associated cell packaging system described here constitute the first retroviral vector system for gene transfer based on a likely human gammaretrovirus, with particular tropism for human cells and tissues. Our results suggest that the pXC vector is a useful tool for gene transfer in human cells and it is possible that this vector will contribute to elucidation of the interactions of XMRV and its human host. In addition, this new XMRV-derived retroviral vector has the potential to be used for stable transfection of human cells and for preclinical studies of gene therapy.

## Competing interests

The authors declare that they have no competing interests.

## Authors' contributions

DCG participated in gene isolation, plasmid construction, transduction assays, and writing this manuscript. ARM conceived of the study, helped interpret the data and write the manuscript. DC aided in the design and coordination, the interpretation of the data and helped to draft the manuscript. All authors read and approved the final manuscript.
